# Development of a New Index Based on Preoperative Serum Lipocalin 2 to Predict Post-LSG Weight Reduction

**DOI:** 10.1007/s11695-022-05916-1

**Published:** 2022-02-09

**Authors:** Nannan Li, Bei Xu, Jiangping Zeng, Shihui Lei, Lei Gu, Lijin Feng, Bing Zhu, Yueye Huang, Lu Wang, Lili Su, Shen Qu, Xiaoyun Cheng, Le Bu

**Affiliations:** 1 Department of Endocrinology and Metabolism, Shanghai Tenth People’s Hospital, Tongji University School of Medicine, No. 301 Middle Yanchang Road, Shanghai, 200072 China; 2 Department of Gastrointestinal Surgery, Shanghai Tenth People’s Hospital, Tongji University School of Medicine, No. 301 Middle Yanchang Road, Shanghai, 200072 China; 3 Department of Pathology, Shanghai Tenth People’s Hospital, Tongji University School of Medicine, No. 301 Middle Yanchang Road, Shanghai, 200072 China

**Keywords:** Lipocalin-2, Bariatric surgery, Obesity, Weight loss

## Abstract

**Background:**

Bariatric surgery is the most effective therapy for obesity, but targeted weight reduction is not always achieved. Serum lipocalin-2 (LCN2) is closely associated with obesity, but its impact on weight loss after surgery is unknown. We aimed to access the reliability of LCN2 levels and other parameters as effective predictors of excellent weight loss (≥ 75% excess weight loss (EWL)) 1 year after bariatric surgery.

**Methods:**

This retrospective study evaluated 450 patients (aged 18–65 years) with obesity at 3 months and 1 year after laparoscopic sleeve gastrectomy (LSG) surgery. Seventy-four patients who underwent LSG surgery and met the inclusion and exclusion criteria were included in this study. Serum LCN2, thyroid function, and metabolic and anthropometric parameters were assessed. Weight reduction was expressed as %EWL and percent total weight loss (%TWL) at 3 months and 1 year post surgery. Multivariable logistic regression analysis and receiver operating characteristic (ROC) curve analysis were used to evaluate predictors of ≥ 75%EWL.

**Results:**

In our cohort, %EWL and %TWL were both strongly associated with preoperative serum LCN2 levels. The binary logistic regression analysis showed that preoperative LCN2, waist circumference, and glycated hemoglobin were independent predictors of excellent weight loss.

**Conclusions:**

Based on these results, we determined a new P index with better predictive value for excellent weight reduction (≥ 75%EWL) 1 year after LSG surgery.

**Graphical abstract:**

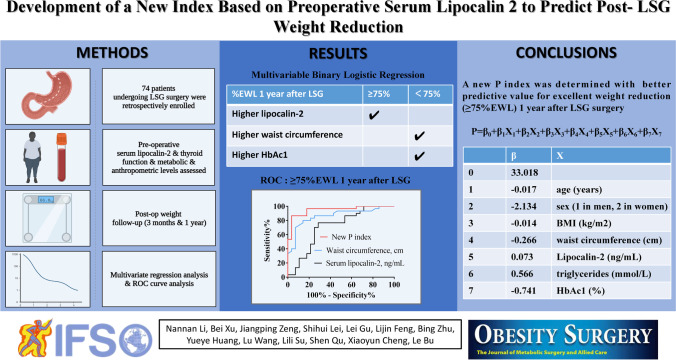

## Introduction

Obesity is associated with many severe comorbidities and has developed into an ongoing pandemic [1]. Bariatric surgery is an effective therapy for rapid, substantial, and durable weight loss, while improving metabolism and reducing mortality and morbidity [2–6]. However, not all bariatric surgery patient reach the expected effective weight loss due to multiple factors [7], such as socioeconomic status, operation style, sex, and preoperative characteristics, which have been studied previously.

Recently, myriad of studies have devoted considerable attention to lipocalin-2 (LCN2) for its potential as a novel therapeutic target for obesity. LCN2 is a new adipokine primarily secreted by osteoblasts and adipocytes. Elevated serum LCN2 levels have been found in obesity models in mice and humans [8, 9]. Furthermore, the involvement of LCN2 might result in the deceleration of spontaneous age-related adiposity by the browning of white adipose tissue and promotion of thermogenic and mitochondrial activity in mice [10]. As reported by Petropoulou et al., LCN2 is a centrally acting anorexigenic hormone that is conserved in humans and non-human primates [11].

These observations revealed that LCN2 plays an indispensable role in obesity. However, the relationship between LCN2 and the remission of obesity after bariatric surgery remain unclear. Our study explored the association of weight reduction after laparoscopic sleeve gastrectomy (LSG) with serum LCN2 levels or other preoperative factors. We propose a new P index to be used as an easy “rule of thumb” for predicting excellent weight reduction after bariatric surgery.

## Materials and Methods

### Subjects

**Fig. 1:**
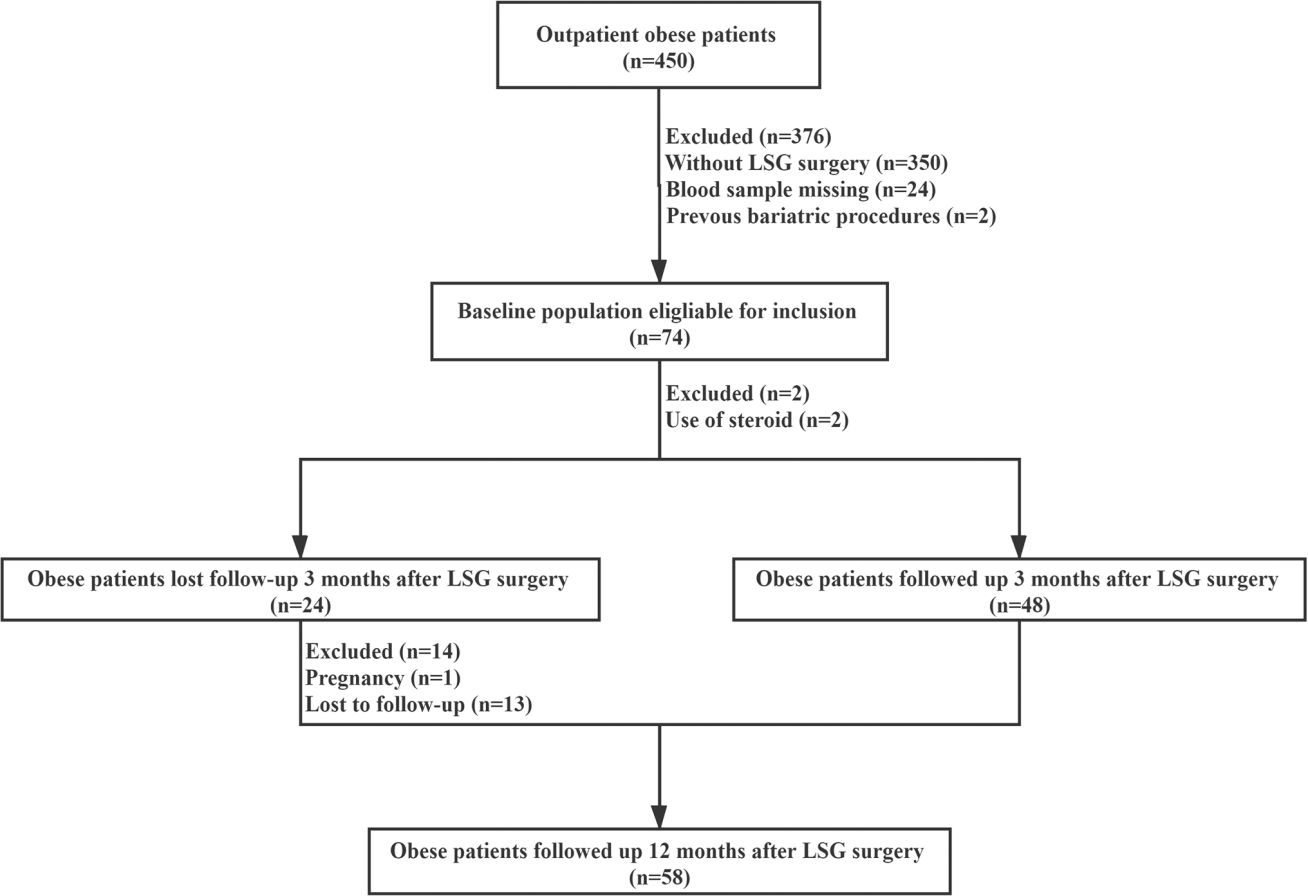
Follow-up study flow chart on patients with obesity subjected to laparoscopic sleeve gastrectomy (LSG) surgery at 3 and 12 months

This retrospective study evaluated 450 patients (aged 18–65 years) with obesity who underwent LSG at Shanghai Tenth People’s Hospital between July 2018 and July 2021. The study recruitment flow chart is shown in Fig. 1. Key inclusion criteria included (1) body mass index (BMI) of 32.5 kg/m^2^ or higher or (2) 27.5 kg/m^2^ or higher BMI with no less than two obesity-related comorbid conditions (e.g., type 2 diabetes, hypertension, dyslipidemia, and obstructive sleep apnea) [9, 12, 13]. Key exclusion criteria included missing data on the required parameters (anthropometric or metabolic data), previous bariatric procedures, use of psychiatric medications or steroids known to affect body weight, severe diseases (malignancy, connective tissue diseases, endocrine diseases, end-stage cardiac, hepatic, or renal failure), and uncontrolled psychiatric diseases [14, 15]. Seventy-four patients who underwent LSG and met the inclusion and exclusion criteria were recruited for this study (Fig. 1). Of the 74 patients, 48 (64.9%) completed follow-up at 3 months and 58 (78.4%) completed follow-up at 12 months after LSG.

The study was approved by the ethics committee of Shanghai Tenth People’s Hospital. All patients provided written informed consent.

### Evaluation of Anthropometric Parameters

Sex, age, body weight, BMI, neck circumference, waist circumference (WC), hip circumference, and blood pressure were evaluated as preoperative parameters on standard medical scales. Weight loss was evaluated by percent excess weight loss (%EWL) and percent total weight loss (%TWL), with the primary outcome of the study being %EWL 1 year after LSG. %EWL after 3 months and 1 year follow-up was calculated by the formula: ((preoperative weight − weight at third / 12th month) / (preoperative weight − ideal weight to produce BMI 24 kg/m^2^) × 100) [16]; %TWL after 3 months and 1 year follow-up was defined by the formula: ((preoperative weight − weight at third / 12th month) / (preoperative weight) × 100). According to Reinhold’s classification [17, 18], insufficient weight loss after LSG surgery was regarded as < 50% EWL, while excellent weight loss was defined when the %EWL was ≥ 75%. Hypertension was defined as blood pressure ≥ 140/90 mmHg, or previously physician-diagnosed and treated [7].

### Measurement of Metabolic Parameters

The serum levels of triglycerides (TG), total cholesterol (TC), free fatty acid (FFA), high-density lipoprotein (HDL) cholesterol, low-density lipoprotein (LDL) cholesterol, glycated hemoglobin (HbA1c), fasting plasma glucose (FPG), and 2-h postprandial plasma glucose (2hPG) were measured and recorded at baseline. Diabetes was defined by FPG ≥ 7.0 mmol/L, HbA1c ≥ 6.5%, 2hPG ≥ 11.1 mmol/L, or previously diagnosed as diabetic and treated; Dyslipidemia was defined by LDL ≥ 4.14 mmol/L, HDL < 1.04 mmol/L, TG ≥ 2.26 mmol/L, or previously diagnosed and treated [7]. Thyroid-stimulating hormone (TSH), free triiodothyronine (FT3), free thyroxine (FT4), total triiodothyronine (TT3), and total thyroxine (TT4) levels were evaluated to determine preoperative thyroid function in patients with obesity.

### ELISA for Serum LCN2 Measurements

Serum LCN2 levels were measured preoperatively using Human Lipocalin-2/NGAL Quantikine ELISA Kit (R&D Systems, Catalog #DLCN20; RRID: AB_2894833). The within-assay (percent coefficient of variation: 3.1–4.4%) and between-assay (5.6–7.9%) variability showed a high level of assay precision.

### Statistics Analysis

Data are presented as mean ± standard deviation (SD) for continuous variables and as percentages for categorical variables. The correlations between serum LCN2 levels and other variables at baseline were assessed using a partial correlation analysis. We conducted Pearson’s or Spearman’s correlation analysis between preoperative variables and %EWL/%TWL to confirm the eligibility for inclusion in the multivariable linear regression analysis, by which the independent factors were determined. We then performed binary logistic regression in Models 1 and 2 to identify independent predictors related to excellent weight loss after LSG surgery, which was generally defined as ≥ 75%EWL 1 year after LSG surgery. Model 1 incorporated all independent factors and adjusted for potential confounding factors, including sex, age, and preoperative BMI; Model 2 included all the covariates in Model 1 and preoperative HbAc1 and TG levels. Ultimately, the receiver operating characteristic (ROC) curve was performed to test the prediction of preoperative LCN2 or waist circumference alone, and Model 2 for the binary outcome, determining “ ≥ 75%EWL” or “ < 75%EWL” 1 year after LSG surgery, and further to identify the optimum cutoff according to the largest Youden index. Statistical significance was set at *P* < 0.05. All statistical analyses were performed using the SPSS Statistics software version 20.0.

## Results

### Baseline Characteristics and Correlation of Preoperative LCN2 Levels with Baseline Variables

Of the 74 subjects (50 female and 24 male) who were included in this study, the average age, baseline BMI, and sex were 32.05 years and 38.69 kg/m^2^; 67.60% female, respectively (Table 1). The baseline population consisted of subjects who were diagnosed with dyslipidemia (66.20%), hypertension (37.80%), and diabetes (54.10%). All subjects exhibited normal thyroid function and the mean serum LCN2 level was 107.50 ng/mL.

**Table 1:** Clinical characteristic of 74 subjects and correlation between serum lipocalin-2 levels and other parameters

Characteristics	value	*r*1	*P*1	*r*2	*P*2
Lipocalin-2, ng/mL	107.50 ± 41.49	-	-	-	-
Age, years	32.05 ± 9.90	-	-	-	-
Gender female (*n*, %)	50.00, 67.60	-	-	-	-
Body mass index, kg/m^2^	38.69 ± 5.96	0.275	0.019^*^	-	-
Weight, kg	110.84 ± 25.44	0.329	0.005^**^	0.197	0.100
Neck circumference, cm	41.92 ± 4.37	0.038	0.752	− 0.162	0.176
Waist circumference, cm	118.22 ± 14.71	0.285	0.015^*^	0.108	0.370
Hip circumference, cm	121.46 ± 12.65	0.256	0.030^*^	0.036	0.767
Hypertension (*n*, %)	28.00, 37.80	-	-	-	-
Dyslipidemia (*n*, %)	49.00, 66.20	-	-	-	-
Triglycerides, mmol/L	2.21 ± 1.38	− 0.078	0.515	− 0.028	0.815
Total cholesterol, mmol/L	4.80 ± 1.01	− 0.254	0.032^*^	− 0.251	0.034^*^
Free fat acid, mmol/L	0.92 ± 2.18	− 0.124	0.300	− 0.234	0.049^*^
HDL cholesterol, mmol/L	1.02 ± 0.24	0.063	0.597	0.101	0.404
LDL cholesterol, mmol/L	2.76 ± 0.82	− 0.177	0.138	− 0.164	0.171
Diabetes (*n*, %)	40.00, 54.10	-	-	-	-
HbA1c, %	7.04 ± 2.12	0.175	0.142	0.167	0.165
Fasting plasma glucose, mmol/L	6.98 ± 2.82	0.120	0.315	0.089	0.463
2-h plasma glucose, mmol/L	11.13 ± 5.25	0.068	0.571	0.068	0.575
Free T3, pmol/L	5.04 ± 0.62	0.028	0.818	0.005	0.964
Free T4, pmol/L	16.60 ± 2.29	0.125	0.294	0.119	0.323
Total T3, nmol/L	1.79 ± 0.37	0.116	0.332	0.019	0.876
Total T4, nmol/L	105.86 ± 21.10	0.123	0.305	0.077	0.525
TSH, mIU/L	2.84 ± 3.19	0.005	0.967	0.007	0.951

1 adjusted for sex and age. 2 adjusted for sex, age, and BMI. *P < 0.05; **P < 0.01; ***P < 0.001

After adjustment for sex and age, we observed that preoperative LCN2 levels were positively associated with BMI, weight, waist circumference, and hip circumference (Table 1). A negative correlation was observed between serum LCN2 levels and total cholesterol at baseline. Notably, even after adjusting for BMI, LCN2 remained significantly negatively correlated with total cholesterol and free fatty acids.

### Association of Weight Reduction with Preoperative LCN2 and Other Parameters

**Fig. 2:**
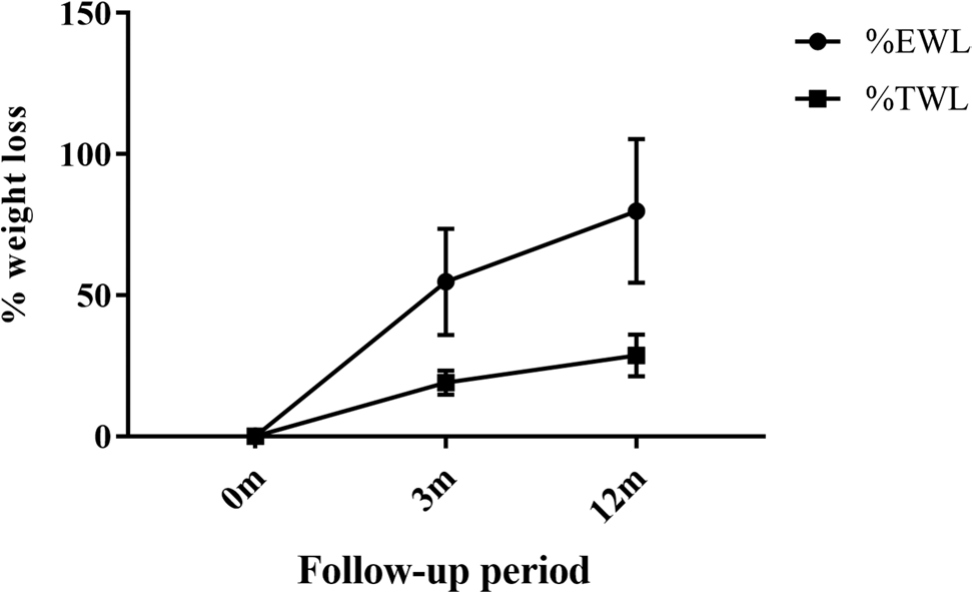
Weight loss plot over time. Values are shown as the mean values of %EWL by the circle dots and %TWL by the square dots, and standard deviation of both by vertical lines. Percent extra weight loss (%EWL) was calculated by the formula: ((preoperative weight − current weight) / (preoperative weight − ideal weight to produce BMI 24 kg/m^2^) × 100); percent total weight loss (%TWL) was defined by the formula: ((preoperative weight − current weight) / (preoperative weight) × 100). 0 m baseline, 3 m 3-month follow-up after laparoscopic sleeve gastrectomy (LSG) surgery, 12 m 12-month follow-up after LSG surgery

Forty-eight patients completed a 3-month follow-up and retained a mean loss of 54.76% of their excess weight and 19.09% of their total weight (Fig. 2). One year after surgery, the patients’ weight loss reached a peak of 79.86% EWL and 28.68% TWL. As shown in Table 2, all five baseline anthropometric parameters (weight, BMI, neck, waist, and hip circumference) were negatively correlated with %EWL at the 3- and 12-month follow-up visits. Free fatty acid and total T3 levels were also negatively correlated with %EWL.

**Table 2:** Associations of weight reduction parameters during follow-up with pre-operative anthropometric and metabolic parameters

Characteristics	3 months				12 months			
	%EWL, %		%TWL, %		%EWL, %		%TWL, %	
	−	*P*	−	*P*	−	*P*	−	*P*
Weight, kg	− 0.625	< 0.001^***^	− 0.139	0.347	− 0.508	< 0.001^***^	0.029	0.831
Body mass index, kg/m^2^	− 0.806	< 0.001^***^	− 0.187	0.203	− 0.649	< 0.001^***^	0.031	0.816
Neck circumference, cm	− 0.431	0.002^**^	− 0.208	0.155	− 0.400	0.002^**^	− 0.061	0.650
Waist circumference, cm	− 0.647	< 0.001^***^	− 0.175	0.235	− 0.631	< 0.001^***^	− 0.101	0.451
Hip circumference, cm	− 0.698	< 0.001^***^	− 0.244	0.094	− 0.592	< 0.001^***^	− 0.053	0.693
Triglycerides, mmol/L	0.106	0.472	0.008	0.956	− 0.002	0.986	− 0.054	0.689
Total cholesterol, mmol/L	− 0.004	0.977	0.019	0.896	0.033	0.803	0.101	0.453
Free fat acid, mmol/L	− 0.461	< 0.001^***^	− 0.176	0.230	− 0.330	0.011^*^	0.059	0.661
HDL cholesterol, mmol/L	0.155	0.294	0.071	0.634	0.162	0.226	0.098	0.464
LDL cholesterol, mmol/L	− 0.087	0.557	− 0.074	0.615	0.052	0.700	0.153	0.250
HbA1c, %	− 0.039	0.794	− 0.009	0.951	− 0.086	0.520	− 0.039	0.769
Fasting plasma glucose, mmol/L	− 0.010	0.949	− 0.004	0.980	− 0.070	0.604	− 0.026	0.849
2-h plasma glucose, mmol/L	0.091	0.536	0.027	0.858	− 0.031	0.820	− 0.066	0.625
Free T3, pmol/L	− 0.062	0.675	0.044	0.766	− 0.034	0.800	0.074	0.580
Free T4, pmol/L	0.049	0.742	− 0.075	0.611	− 0.067	0.617	− 0.121	0.365
Total T3, nmol/L	− 0.371	0.009^**^	− 0.204	0.164	− 0.268	0.042^*^	− 0.045	0.739
Total T4, nmol/L	− 0.019	0.899	0.031	0.834	− 0.034	0.801	0.062	0.642
TSH, mIU/L	− 0.053	0.722	0.003	0.986	0.032	0.811	− 0.064	0.631
Lipocalin-2, ng/mL	0.356	0.013^*^	0.546	< 0.001^***^	0.395	0.002^**^	0.493	< 0.001^***^

*%EWL* percent extra weight loss, *%TWL* percent total weight loss. *P < 0.05; **P < 0.01; ***P < 0.00

Preoperative LCN2 levels were positively associated with both %EWL and %TWL 3-month and 1-year after surgery (Table 2). As summarized in Table 3, multiple linear regression analysis showed that baseline BMI and serum LCN2 levels were independently associated with %EWL at 3-month post-surgery. Furthermore, waist circumference and serum LCN2 levels were independently correlated with %EWL at the first-year follow-up visit.

**Table 3:** Multiple linear regression of percent excess weight loss vs. demographic and clinical variables

%EWL (%)	3 months (*R*^2^ = 0.710)		12 months (*R*^2^ = 0.614)	
	*β*	*P* value	*β*	*P* value
Age	− 0.095	0.320	0.011	0.907
Sex (female vs. male)	− 0.043	0.686	− 0.055	0.616
Body mass index	− 0.624	< 0.001^***^	− 0.302	0.072
Waist circumference	− 0.176	0.308	− 0.449	0.015^*^
Free fat acid	0.146	0.142	0.059	0.556
Total T3	− 0.097	0.343	0.036	0.729
Lipocalin-2	0.385	< 0.001^***^	0.420	< 0.001^***^

Two multiple liner regression models were provided. Percent excess weight loss (%EWL) was the independent variable, and *R*^2^ was the percentage of %EWL explained by the whole model. *P < 0.05; **P < 0.01; ***P < 0.001

### Preoperative LCN2, Waist Circumference, and HbAc1 Were Independent Predictors for Excellent Weight Loss

Excellent weight loss was defined as an EWL ≥ 75% 1 year after LSG surgery. Of the 58 subjects who underwent LSG surgery and were incorporated into a logistic regression analysis, 28 patients did not achieve excellent weight loss. In multiple analyses, as shown in Table 4 Model 1 (Nagelkerke *R*^2^ = 0.673), lower waist circumference and higher serum LCN2 levels at baseline were independently associated with excellent weight loss after LSG surgery. After adjusting for preoperative HbAc1 and TG, preoperative HbAc1 levels indicated an independent association with excellent weight loss (Model 2, Nagelkerke *R*^2^ = 0.739). Therefore, preoperative waist circumference and serum LCN2 and HbAc1 levels might independently predict excellent weight loss 1-year post-operation in our cohort.

**Table 4:** Variables significantly affecting a response of more than 75% excess weight loss after LSG surgery

	Model 1		Model 2	
	Nagelkerke *R*^2^ = 0.673		Nagelkerke *R*^2^ = 0.739	
	*OR* (95% *CI*)	*P* value	*OR* (95% *CI*)	*P* value
Age	0.979 (0.910, 1.053)	0.563	0.983 (0.905, 1.067)	0.681
Sex (female vs. male)	0.248 (0.022, 2.851)	0.263	0.118 (0.007, 2.077)	0.144
Body mass index	0.910 (0.702, 1.179)	0.475	0.987 (0.720, 1.353)	0.933
Waist circumference	0.837 (0.726, 0.966)	0.015^*^	0.766 (0.625, 0.940)	0.011^*^
Lipocalin-2	1.055 (1.019, 1.092)	0.003^**^	1.076 (1.028, 1.126)	0.002^**^
HbAc1	-	-	0.477 (0.239, 0.952)	0.036^*^
Triglycerides	-	-	1.761 (0.917, 3.383)	0.089

Excellent weight loss and not excellent were defined as EWL ≥ 75% and EWL < 75%, respectively. Adjusted odds ratio (*OR*) and 95% confidence interval (*CI*) for the probability of excellent weight loss 1 year after LSG surgery were given. *P < 0.05; **P < 0.01; ***P < 0.001

#### A New P Index as a Predictive Model for Excellent Weight Reduction

**Fig. 3:**
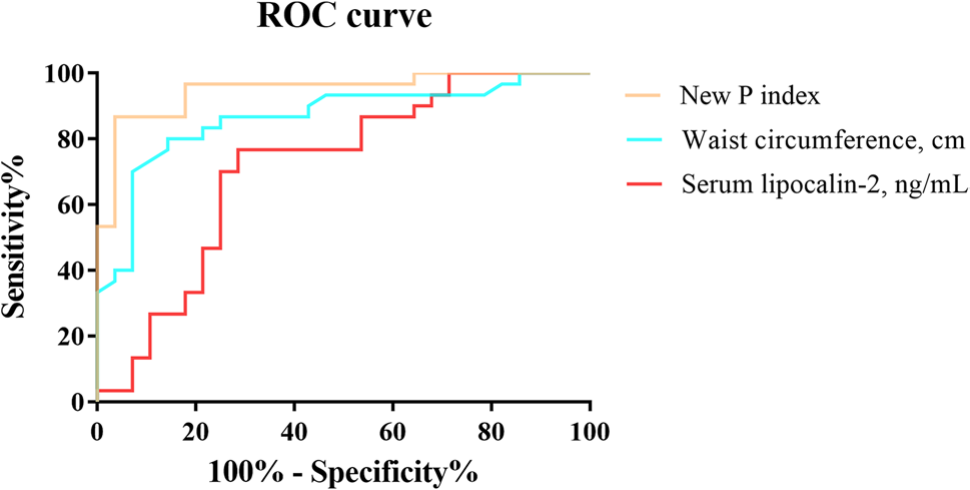
Receiver operating characteristic (ROC) curve for serum lipocalin-2 (red line), waist circumference (blue line), and new P index (orange line) respectively in the prediction of a surgery response of ≥ 75% EWL 1 year after LSG surgery

Based on our findings, we identified waist circumference and preoperative LCN2 as the most predictive parameters for excellent weight reduction 12-month post-surgery in our dataset. Both variables achieved acceptable performance in ROC analyses with optimal cutoffs for waist circumference at 117.647 cm (sensitivity 80.00%, specificity 85.70%) and LCN2 level at 103.15 ng/mL (sensitivity 76.70%, specificity 71.40%), respectively (Table 5 and Fig. 3).

**Table 5:** Receiver-operating characteristic (ROC) for lipocalin-2, waist circumference, and the new P index as predictors for a surgery response of ≥ 75% EWL 1 year after LSG surgery

	Optimized cutoff value	Sensitivity (%)	Specificity (%)	Youden index	AUC	Standard error (SE)	95%CI	*P* value
New index P	0.649	86.700	96.400	0.831	0.949	0.029	0.893-1.000	< 0.001^***^
Waist circumference, cm	117.647	80.000	85.700	0.657	0.865	0.050	0.767-0.962	< 0.001^***^
Lipocalin-2, ng/mL	103.150	76.700	71.400	0.481	0.715	0.070	0.578-0.853	0.005^**^

New P index was calculated by the formula: - 0.017×age (years) - 2.134 × sex - 0.014 × BMI (kg/m^2^) - 0.266 × WC (cm) + 0.073 × LCN2 (ng/mL) + 0.566 × triglycerides (mmol/L) - 0.741 × HbAc1 (%) +33.018]. Values were presented as 1 in male sex and 2 in female sex, and WC as waist circumference.

*AUC *area under the curve, *95%CI* 95% confidence interval.

*P < 0.05; **P < 0.01; ***P < 0.001

A new index, P, was defined by Model 2 (Table 5 and Fig. 3). The new equation was developed as follows: P index = (− 0.017 × age (years) − 2.134 × sex − 0.014 × BMI (kg/m^2^) − 0.266 × waist circumference (cm) + 0.073 × LCN2 (ng/mL) + 0.566 × triglycerides (mmol/L) − 0.741 × HbAc1 (%) + 33.018), with sex referred to as 1 in men and 2 in women. The AUC of the new P index was 0.949, significantly larger than those of LCN2 and waist circumference, indicating that the new P index is more reliable for predicting 1-year weight reduction after the operation. The optimum cutoff point for the new P index at 0.649 predicted excellent weight loss 12-month post-surgery with a sensitivity of 86.70% and specificity of 96.40%, both parameters were higher than those achieved with waist circumference or serum LCN2 alone.

## Discussion

Bariatric surgery is the only practical and effective intervention alternative for most patients with extreme obesity to achieve weight loss [19]. However, the effectiveness of weight loss varies among patients. According to Reinhold’s classification [17, 18], success was regarded as ≥ 50% EWL, while excellent weight loss was defined when the %EWL was ≥ 75%. Some physiological characteristics of preoperative patients have been studied to predict success (%EWL ≥ 50%) after bariatric surgery, such as sex, age, BMI, waist circumference, type II diabetes, dyslipidemia, hypertension, and smoking, with a rough similarity but lack of consensus [20]. Although ≥ 75% EWL was associated with better metabolic syndrome remission 1 year after bariatric surgery [21], there are few published studies regarding predictors of excellence (%EWL ≥ 75%) after bariatric surgery, LCN2 is closely associated with obesity and obesity-related metabolic disorders, but its impact on weight loss after surgery is unknown. Our study aimed to ascertain whether preoperative serum LCN2 or any other factors independently predict excellence (%EWL ≥ 75%) in weight reduction after LSG surgery.

Our previous observations indicated that patients with obesity had elevated serum LCN2 levels [8, 9]. In our study, excellent weight loss (%EWL ≥ 75%) at 1-year follow-up after LSG surgery was independently correlated with higher preoperative LCN2 levels. In addition, %TWL was also significantly associated with serum LCN2, indicating that patients with higher preoperative LCN2 levels tend to lose more initial weight and are more likely to reach their ideal weight. Our study suggests that elevated serum LCN2 levels in patients with obesity might serve as a biomarker or even play an important role in weight reduction after LSG surgery. In a previous study, the ablation of LCN2 profoundly impaired adaptive thermogenesis through suppression of brown adipose tissue activity [22–24]. Circulating LCN2 suppresses food intake by activating the melanocortin-4 receptor signaling pathway in the hypothalamus [25]. LCN2 seems to be a key factor linking energy intake and energy expenditure with obesity, partly explaining the excellent weight reduction in patients with higher LCN2 at baseline. However, an in-depth understanding of these mechanisms remains to be elucidated.

In our cohort, a cross-sectional study showed that serum LCN2 levels were associated with several adiposity variables, including BMI, weight, waist circumference, and hip circumference, which concurs with a previous study in 229 adults, suggesting that increased fat mass might partly account for elevated serum LCN2 levels in humans with obesity [26]. Furthermore, we found a significantly negative association between serum LCN2 levels and several variables correlated with adverse lipid profiles. In agreement with the animal data, our findings implied that LCN2 might be a protective factor against lipid metabolic dysregulation [22, 27].

In addition to preoperative serum LCN2 levels, we found that waist circumference and HbAc1 levels at baseline were independent predictors for %EWL. The effects of BMI and waist circumference remain controversial in predicting the success of bariatric surgery studies [28–30]. We found that %EWL was negatively associated with waist circumference but not with BMI in multiple regression analysis considering BMI and waist circumference together. In binary logistic regression analysis, Model 2 showed that HbAc1 was an independent predictor of excellence after LSG surgery, consistent with a study by Ortega et al. [30].

Previous bariatric surgery studies have focused on the success of surgery (defined as %EWL ≥ 50% or mean minus 1 SD) and performed a multiple logistic regression analysis including variables such as anthropometric and metabolic index, surgery style, and surgery access to predict weight reduction after bariatric surgery [28, 30–32]. In addition, several novel predictors (e.g., serum asprosin, phase angle, and apnea–hypopnea index) of weight reduction efficacy have emerged in recent years, but none of them, when combined with other parameters, provide a predictive model or display significance in a model [7, 15, 20, 33, 34]. To our knowledge, this is the first study to focus on preoperative predictors of excellent weight reduction (defined as %EWL ≥ 75%) after LSG surgery, pointing to better weight reduction and better metabolic relief. In our study, we determined Model 2 as a new P index considering serum LCN2 and other variables including sex, age, adiposity, and glucolipid metabolism. The optimum cutoff of P of 0.649 showed a high sensitivity of 86.70% and specificity of 96.40% for predicting excellent weight reduction post-surgery.

Our study had a few limitations. The main limitation is the relatively small single-center dataset, which limited the statistical power of the results, as well as the lack of a validation cohort. Second, the follow-up period was relatively short (1 year), and the follow-up rate at 3 months is only 64.9%. Additionally, because the cohort included only Chinese individuals, any findings may not be generalizable to other ethnic groups. Additionally, we cannot exclude the effect of any unmeasured bias or confounding factors. Future prospective studies may provide more reliable information on the prediction of post-surgery weight loss. We have proposed a non-validated prediction model that needs to be confirmed in a large prospective study. The problem of excellent weight loss is multifactorial, and its resolution will require more research in the future to uncover more predictors to provide an optimal prediction model.

## Conclusion

In this study, we have provided a better model and a new P index based on preoperative serum LCN2 and other parameters as effective predictors for excellent weight reduction during the 1-year postoperative follow-up after bariatric surgery. Our model and the proposed index still require future studies to confirm their external validity, clinical applicability, and generalizability.

## References

[1] Gastroenterology TL, Obesity H (2021). another ongoing pandemic. lancet Gastroenterol hepatol.

[2] Maggard-Gibbons M, Maglione M, Livhits M, Ewing B, Maher AR, Hu J (2013). Bariatric surgery for weight loss and glycemic control in nonmorbidly obese adults with diabetes: a systematic review. JAMA.

[3] Müller-Stich BP, Senft JD, Warschkow R, Kenngott HG, Billeter AT, Vit G (2015). Surgical versus medical treatment of type 2 diabetes mellitus in nonseverely obese patients: a systematic review and meta-analysis. Ann Surg.

[4] Panagiotou OA, Markozannes G, Adam GP, Kowalski R, Gazula A, Di M (2018). Comparative effectiveness and safety of bariatric procedures in medicare-eligible patients: a systematic review. JAMA surgery..

[5] O’Brien PE, Hindle A, Brennan L, Skinner S, Burton P, Smith A (2019). Long-term outcomes after bariatric surgery: a systematic review and meta-analysis of weight loss at 10 or more years for all bariatric procedures and a single-centre review of 20-year outcomes after adjustable gastric banding. Obes Surg.

[6] Sjöström L, Peltonen M, Jacobson P, Sjöström CD, Karason K, Wedel H (2012). Bariatric surgery and long-term cardiovascular events. JAMA.

[7] Borges-Canha M, Neves JS, Mendonça F, Silva MM, Costa C, P MC (2021). Beta cell function as a baseline predictor of weight loss after bariatric surgery. Frontiers endocrinol.

[8] Mosialou I, Shikhel S, Luo N, Petropoulou PI, Panitsas K, Bisikirska B, et al. Lipocalin-2 counteracts metabolic dysregulation in obesity and diabetes. The Journal of experimental medicine. 2020;217(10). 10.1084/jem.20191261 PMC7537391 32639539

[9] Wen X, Zhu B, Zhang Y, Mei F, Cheng X, Qian C (2019). Alterations in fat mass and bone mineral density are associated with decreased lipocalin-2 after laparoscopic sleeve gastrectomy in obese Chinese women. Obes Surg.

[10] Meyers K, López M, Ho J, Wills S, Rayalam S, Taval S (2020). Lipocalin-2 deficiency may predispose to the progression of spontaneous age-related adiposity in mice. Sci Rep.

[11] Petropoulou PI, Mosialou I, Shikhel S, Hao L, Panitsas K, Bisikirska B, et al. Lipocalin-2 is an anorexigenic signal in primates. eLife. 2020;9. 10.7554/eLife.58949 PMC7685704 33231171

[12] Robert M, Espalieu P, Pelascini E, Caiazzo R, Sterkers A, Khamphommala L (2019). Efficacy and safety of one anastomosis gastric bypass versus Roux-en-Y gastric bypass for obesity (YOMEGA): a multicentre, randomised, open-label, non-inferiority trial. Lancet (London, England).

[13] Ke Z, Li F, Gao Y, Tan D, Sun F, Zhou X (2021). The use of visceral adiposity index to predict diabetes remission in low BMI Chinese patients after bariatric surgery. Obes Surg.

[14] Rebelos E, Moriconi D, Scalese M, Denoth F, Molinaro S, Siciliano V (2020). Impact of postprandial hypoglycemia on weight loss after bariatric surgery. Obes Surg.

[15] Wang CY, Lin TA, Liu KH, Liao CH, Liu YY, Wu VC (2019). Serum asprosin levels and bariatric surgery outcomes in obese adults. Int j obesity (2005).

[16] Zhou B. [Predictive values of body mass index and waist circumference to risk factors of related diseases in Chinese adult population]. Zhonghua liu xing bing xue za zhi = Zhonghua liuxingbingxue zazhi. 2002;23(1):5–10. 12015100

[17] Carandina S, Soprani A, Zulian V, Cady J (2021). Long-term results of one anastomosis gastric bypass: a single center experience with a minimum follow-up of 10 years. Obes Surg.

[18] Reinhold RB (1982). Critical analysis of long term weight loss following gastric bypass. Surgery, gynecology & obstetrics.

[19] Buchwald H, Avidor Y, Braunwald E, Jensen MD, Pories W, Fahrbach K (2004). Bariatric surgery: a systematic review and meta-analysis. JAMA.

[20] Gerken ALH, Rohr-Kräutle KK, Weiss C, Seyfried S, Reissfelder C, Vassilev G (2021). Handgrip strength and phase angle predict outcome after bariatric surgery. Obes Surg.

[21] Tu Y, Pan Y, Han J, Pan J, Zhang P, Jia W (2021). A total weight loss of 25% shows better predictivity in evaluating the efficiency of bariatric surgery. Int j obesity (2005).

[22] Guo H, Jin D, Zhang Y, Wright W, Bazuine M, Brockman DA (2010). Lipocalin-2 deficiency impairs thermogenesis and potentiates diet-induced insulin resistance in mice. Diabetes.

[23] Guo H, Bazuine M, Jin D, Huang MM, Cushman SW, Chen X (2013). Evidence for the regulatory role of lipocalin 2 in high-fat diet-induced adipose tissue remodeling in male mice. Endocrinology.

[24] Zhang Y, Guo H, Deis JA, Mashek MG, Zhao M, Ariyakumar D (2014). Lipocalin 2 regulates brown fat activation via a nonadrenergic activation mechanism. J Biol Chem.

[25] Mosialou I, Shikhel S, Liu JM, Maurizi A, Luo N, He Z (2017). MC4R-dependent suppression of appetite by bone-derived lipocalin 2. Nature.

[26] Wang Y, Lam KSL, Kraegen EW, Sweeney G, Zhang J, Tso AW (2007). Lipocalin-2 is an inflammatory marker closely associated with obesity, insulin resistance, and hyperglycemia in humans. Clin Chem.

[27] Deis JA, Guo H, Wu Y, Liu C, Bernlohr DA, Chen X (2019). Adipose lipocalin 2 overexpression protects against age-related decline in thermogenic function of adipose tissue and metabolic deterioration. Molecular metabolism.

[28] Barhouch AS, Padoin AV, Casagrande DS, Chatkin R, Süssenbach SP, Pufal MA (2016). Predictors of excess weight loss in obese patients after gastric bypass: a 60-month follow-up. Obes Surg.

[29] Menenakos E, Stamou KM, Albanopoulos K, Papailiou J, Theodorou D, Leandros E (2010). Laparoscopic sleeve gastrectomy performed with intent to treat morbid obesity: a prospective single-center study of 261 patients with a median follow-up of 1 year. Obes Surg.

[30] Ortega E, Morínigo R, Flores L, Moize V, Rios M, Lacy AM (2012). Predictive factors of excess body weight loss 1 year after laparoscopic bariatric surgery. Surg Endosc.

[31] Coupaye M, Sabaté JM, Castel B, Jouet P, Clérici C, Msika S (2010). Predictive factors of weight loss 1 year after laparoscopic gastric bypass in obese patients. Obes Surg.

[32] Sisik A, Basak F (2020). Presurgical predictive factors of excess weight loss after laparoscopic sleeve gastrectomy. Obes Surg.

[33] de Raaff CA, Coblijn UK, de Vries N, Heymans MW, van den Berg BT, van Tets WF (2016). Predictive factors for insufficient weight loss after bariatric surgery: does obstructive sleep apnea influence weight loss?. Obes Surg.

[34] Neves JS, Souteiro P, Oliveira SC, Pedro J, Magalhães D, Guerreiro V (2019). Preoperative thyroid function and weight loss after bariatric surgery. Int j obesity (2005).

